# Evaluation of environmental scanning electron microscopy for analysis of *Proteus mirabilis* crystalline biofilms *in situ* on urinary catheters

**DOI:** 10.1111/1574-6968.12451

**Published:** 2014-05-12

**Authors:** Nina Holling, Cinzia Dedi, Caroline E Jones, Joseph A Hawthorne, Geoffrey W Hanlon, Jonathan P Salvage, Bhavik A Patel, Lara M Barnes, Brian V Jones

**Affiliations:** 1 School of Pharmacy and Biomolecular Sciences, University of BrightonEast Sussex, UK; 2 Queen Victoria Hospital NHS Foundation TrustEast Grinstead, West Sussex, UK

**Keywords:** *Proteus mirabilis*, crystalline biofilm, environmental scanning electron microscopy, catheter-associated urinary tract infection

## Abstract

*Proteus mirabilis* is a common cause of catheter-associated urinary tract infections and frequently leads to blockage of catheters due to crystalline biofilm formation. Scanning electron microscopy (SEM) has proven to be a valuable tool in the study of these unusual biofilms, but entails laborious sample preparation that can introduce artefacts, undermining the investigation of biofilm development. In contrast, environmental scanning electron microscopy (ESEM) permits imaging of unprocessed, fully hydrated samples, which may provide much insight into the development of *P. mirabilis* biofilms. Here, we evaluate the utility of ESEM for the study of *P. mirabilis* crystalline biofilms *in situ*, on urinary catheters. In doing so, we compare this to commonly used conventional SEM approaches for sample preparation and imaging. Overall, ESEM provided excellent resolution of biofilms formed on urinary catheters and revealed structures not observed in standard SEM imaging or previously described in other studies of these biofilms. In addition, we show that energy-dispersive X-ray spectroscopy (EDS) may be employed in conjunction with ESEM to provide information regarding the elemental composition of crystalline structures and demonstrate the potential for ESEM in combination with EDS to constitute a useful tool in exploring the mechanisms underpinning crystalline biofilm formation.

## Introduction

*Proteus mirabilis* poses particular problems for the management of patients undergoing long-term urethral catheterisation, with encrustation and blockage of catheters a hallmark of *P. mirabilis* catheter-associated urinary tract infection (CAUTI; Stickler *et al*., 1993; Stickler & Zimakoff, 1994; Stickler & Morgan, 2008). Encrustation stems from the ability of *P. mirabilis* to form dense biofilm communities on catheter surfaces, and the production of a potent urease enzyme, which converts urea to ammonia and carbon dioxide elevating urinary pH (Griffith *et al.*, 1976; Jones & Mobley, 1987; Stickler, 2008). This results in the precipitation of calcium and magnesium phosphates, leading to the formation of crystals of struvite (ammonium magnesium phosphate) and hydroxyapatite (calcium phosphates), which become trapped in the developing biofilm (Griffith *et al.*, 1976; Hedelin *et al.*, 1984; Cox & Hukins, 1989; Stickler & Zimakoff, 1994). The biofilm matrix itself has been shown to enhance crystal formation by attracting and binding magnesium and calcium ions, and stabilising the growth of crystals (Clapham *et al.*, 1990; Dumanski *et al.*, 1994). Ultimately, this process results in the mineralisation of *P. mirabilis* biofilms and the formation of crystalline biofilm structures that encrust catheter surfaces, obstruct urine flow and lead to more serious complications. As such, understanding the processes underlying biofilm formation in *P. mirabilis* will be important in developing effective strategies to prevent or treat CAUTI.

Scanning electron microscopy (SEM) has proven to be a powerful tool for the study of crystalline biofilms, particularly when used in combination with representative models of CAUTI and techniques such as X-ray spectroscopy (Cox & Hukins, 1989; Morris & Stickler, 1998; Stickler *et al.*, 1999; Jones *et al.*, 2005; Stickler & Morgan, 2006; Macleod & Sticker, 2007; Stickler, 2008; Hannig *et al.*, 2010). However, a drawback of SEM is the need to first process samples before viewing, which involves fixation and dehydration, followed by coating in conductive materials such as gold or platinum (Cox & Hukins, 1989; Stickler *et al.*, 1999; Jones *et al.*, 2004; Bergmans *et al.*, 2005; Stickler & Morgan, 2006; Macleod & Sticker, 2007; Hannig *et al.*, 2010). This processing can potentially alter the structure of biofilms and introduce changes or artefacts, with a variety of sample preparation methods available and little standardisation between studies (Hannig *et al.*, 2010). In the case of crystalline biofilms, the need to also preserve the crystalline component adds further scope for introduction of artefacts or loss of structural details.

In contrast environmental scanning electron microscopy (ESEM) has the potential to image biofilms in their native, hydrated state without the requirement for processing or fixation (Bergmans *et al.*, 2005; Hannig *et al.*, 2010). There is also the possibility of employing analytical techniques such as energy-dispersive X-ray spectroscopy (EDS) in conjunction with ESEM to survey the elemental content of samples, which has clear utility when applied to crystalline biofilms (Stickler *et al.*, 1999; Stickler & Morgan, 2008). However, this approach does not perform well for all sample types (Bergmans *et al.*, 2005; Hannig *et al.*, 2010), and the utility of ESEM in analysis of mature crystalline biofilms remains to be established. Here, we evaluate the use of ESEM and associated EDS for the direct imaging and analysis of mature *P. mirabilis* crystalline biofilms *in situ* on urinary catheters.

## Materials and methods

### Bacterial strains, culture and media

*Proteus mirabilis* B4 is a recent clinical isolate from an encrusted catheter (Jones *et al.*, 2004, 2005). A derivative mini-*Tn*5 mutant, designated NHBFF9 and characterised in previous studies (Holling *et al.*, 2014), was also utilised here to evaluate ESEM and EDS in comparing levels of biofilm formation. This mutant was created by the conjugational transfer of the pUTKm2 transposon delivery vector to *P. mirabilis* strain B4 from the *Escherichia coli* S17.1λpir donor strain as previously described (de Lorenzo & Timmis, 1994; Jones *et al.*, 2004). Bacteria were routinely cultured in Luria–Bertani (LB) medium (Fisher Scientific, UK) and supplemented with 50 μg mL^−1^ kanamycin for the routine culture of the *Tn*5 mutant unless stated otherwise. Artificial urine (AU) medium was based on that described by Stickler *et al*. (1999) and composed of sodium di-sulphate 11.5 g L^−1^, magnesium chloride (hexahydrate) 3.25 g L^−1^, sodium chloride 23 g L^−1^, tri-sodium citrate 3.25 g L^−1^, sodium oxalate 0.1 g L^−1^, potassium di-hydrogen orthophosphate 14 g L^−1^, potassium chloride 8 g L^−1^, ammonium chloride 5 g L^−1^, calcium chloride dehydrate 3.25 g L^−1^, urea 125 g L^−1^, gelatine 25 g L^−1^ (Fisher Scientific) and tryptone soya broth (Oxoid) 5 g L^−1^. Stock solutions of urea and calcium chloride dehydrate were sterilised separately by membrane filtration (0.45 μm; Sartorius, UK) and added to other components (which were sterilised by autoclaving), to provide the final AU medium with all ingredients at concentrations noted above, and a final pH of 6.1.

### *In vitro* bladder models

Bladder models were set up and operated as previously described using size 14Ch all-silicone catheters and AU medium (Stickler *et al.*, 1999; Holling *et al.*, 2014) (Supporting Information, Fig. S1). Bladder models were inoculated with a 10-mL culture at *c*. 10^9^ CFU mL^−1^, and cells allowed to establish for 1 h before flow was initiated at a constant rate of 0.75 mL min^−1^. Viable cells in ‘bladders’ were enumerated at the start and end of experiments by colony counts on LB agar. The pH of urine in ‘bladders’ was also measured at model activation and experimental endpoints. Models were permitted to run until blockage (18–24 h) to develop mature biofilms (wild type only), or for a fixed time (10 h) to permit direct comparisons of biofilms formed between the wild-type and mutant NHBFF9. All bladder model experiments were repeated in triplicate.

### SEM with processed samples

One-centimetre catheter sections directly beneath eyeholes were cut longitudinally to expose the intraluminal crystalline biofilm, before being fixed in 2.5% w/v glutaraldehyde in 0.1 M cacodylate buffer at pH 7.2 overnight. The following day, samples were placed into fresh 0.1 M cacodylate buffer at pH 7.2, prior to dehydration in a series of ethanol solutions (25, 50, 75, 95, 95 and 100% w/v, 1-h incubations in each). Sections were then air-dried and mounted on aluminium stubs using carbon cement (Agar Scientific, Stansted, UK). Samples were viewed using a Zeiss-evo LS15 microscope (Carl Zeiss Ltd, UK), either uncoated in variable pressure (VP) mode, or were subsequently coated with platinum and viewed under high vacuum (HV). Parameters for VP imaging were 100-μm VP aperture, a chamber pressure of 50 pa with 0% humidity, an accelerating voltage of 20 kV EHT and using a VP secondary electron detector. For HV observations, samples were sputter-coated with platinum using a Quorum Q150T ES system (Quorum Technologies, UK), before being viewing at a chamber pressure of *c*. 3.5 × 10^−3^ pa (0% humidity) using an accelerating voltage of 5 kV EHT and a secondary electron detector.

### ESEM of fully hydrated samples

Catheters were dissected as described above for conventional SEM, before being mounted using Tissue-Tek CRYO-OCT compound mountant (Agar Scientific). Stubs were placed onto a liquid-cooled Deben CoolStage at a temperature of *c*. 1.5 °C and viewed using the same Zeiss-evo LS15 microscope (Carl Zeiss Ltd) used for SEM, operating in extended pressure (EP) mode, and using the following parameters and conditions: 100-μm upper EP aperture, 500-μm lower EDS EP aperture, a chamber pressure of 500–560 pa with *c*. 85% humidity, an accelerating voltage of 20 kV EHT and using a five quadrant backscatter detector. EDS spectra from areas of interest were obtained using an 80-mm^2^ X-max silicon drift detector (Oxford instruments, UK). EDS data were analysed using the Aztec software to produce EDS spectra (Oxford instruments).

### Quantification of calcium on catheter sections

The same catheter sections from timed bladder models as analysed by ESEM and EDS were also used to quantify total calcium by flame photometry as previously described (Holling *et al.*, 2014). Following ESEM viewing, catheter sections were submerged in 2 mL of a solution of ammonium oxalate (95% w/v) and oxalic acid (5% w/v), mixed vigorously for 3 min, then incubated at room temperature for 30 min. Catheter sections were then removed, the remaining mixture centrifuged (3000 ***g*** for 10 min) and the supernatant discarded. Pellets were resuspended in 5 mL perchloric acid (0.05 M), and samples thoroughly mixed before a further round of centrifugation (3000 ***g*** for 2 min). Levels of calcium dissolved in supernatants were determined using a flame photometer (Corning, Flame Photometer 410), calibrated using calcium standards at 100, 75, 50 and 25 μg mL^−1^.

### Quantification of biomass on catheter surfaces

For quantification of biomass, catheters removed from bladder models were dissected as for ESEM and processed as described by Holling *et al*. (2014). Sections were transferred to sterile centrifuge tubes and gently rinsed with 5 mL sterile deionised water (SDW) to remove loosely attached or nonadherent debris. Catheter sections were subsequently submerged in 1 mL of 0.5% crystal violet solution (Fisher Scientific) and incubated for 10 min at room temperature. Catheter sections were then transferred to fresh tubes and gently washed three times with SDW to remove excess stain. SDW was completely removed before addition of 1 mL of dimethyl sulfoxide (DMSO; Fisher Scientific). Tubes were then mixed well for 1 min to elute crystal violet dye retained by biofilms. Absorbance of the resulting solutions was measured at 595 nm using a spectrophotometer (Jenway 6300).

### Statistical analysis

All statistical analysis was performed using Prism 6.0c For Mac OS X (GraphPad Software Inc.; www.graphpad.com). Data were analysed using either Student's *t*-test or anova.

## Results

### Comparative analysis of ESEM and SEM for imaging of crystalline biofilms

**Fig 1:**
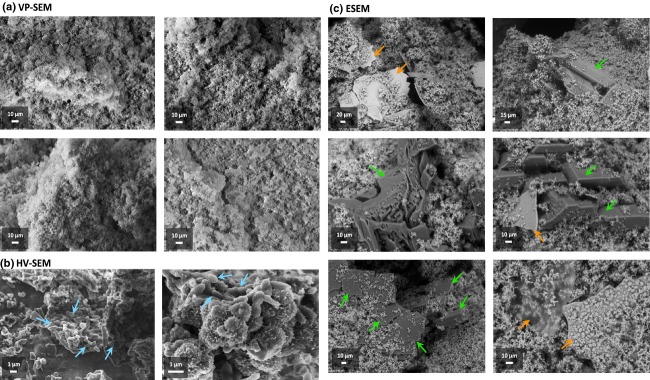
Comparison of conventional SEM approaches and ESEM for observation of *Proteus mirabilis* crystalline biofilms. Images show representative regions of mature biofilms developed on all-silicone Foley catheters using *in vitro* bladder models supplied with a standardised AU medium. (a, b) Images obtained following conventional processing of catheter sections with glutaraldehyde fixation followed by ethanol dehydration and viewing of uncoated samples using VP-SEM, or platinum-coated samples at high magnification using HV-SEM. Arrows indicate regions in HV-SEM images where individual cells are visible (blue arrows). (c) Images obtained from observation of unprocessed biofilms with ESEM. Arrows indicate examples of distinct crystal types designated as either type 1 (green arrows; large electron dense) or type 2 (orange arrows; ‘sheet-like’ structures). Images represent examples from analysis of crystalline biofilms developed in three replicate experiments and derived from distinct catheter biofilms. Images are representative of a minimum of 15 fields of view for each SEM method.

In images from both SEM and ESEM, the bulk biofilm matrix appeared saturated with amorphous crystalline material, which obscured individual cells, consistent with previous SEM observations of *P. mirabilis* catheter biofilms (Winters *et al.*, 1995; Stickler & Morgan, 2008; Fig.1). Individual cells were only visible when more sparsely populated regions of catheter sections, processed for conventional SEM viewing, were viewed under HV and high magnification (Fig.1b). However, a general absence of well-defined crystal structures in processed samples was apparent, and in stark contrast to biofilms observed using ESEM, where large crystals embedded within the biofilm were prevalent features (Fig.1c).

Two distinct crystal types were observed using ESEM, designated type 1 and type 2 (Fig.1c). Type 1 crystals manifest as large electron-dense structures embedded within the bulk biofilm matrix and were congruent with previous descriptions of ammonium magnesium phosphate crystals (struvite; Griffith *et al.*, 1976; Hedelin *et al.*, 1984; Cox & Hukins, 1989; Stickler & Zimakoff, 1994). Type 2 crystals were flat ‘sheet-like’ structures that have not previously been described as components of the mature crystalline biofilm in other studies.

### ESEM and associated EDS analysis of crystalline biofilm composition

**Fig 2:**
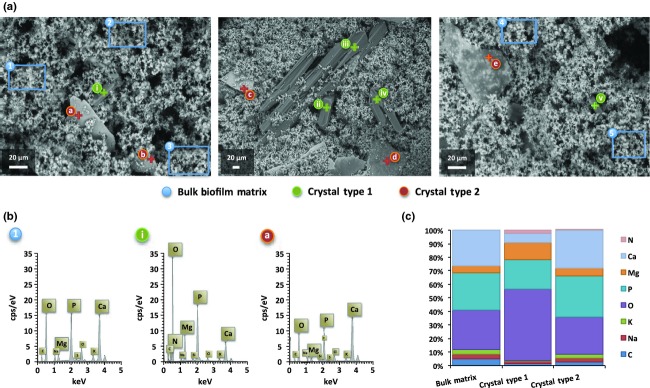
Analysis of crystalline biofilm composition using ESEM and EDS. (a) ESEM images showing regions of biofilms, and crystalline structures subject to EDS analysis. Symbols on images denote structures or regions subject to EDS analysis as described in the associated legend. Blue symbols and boxed regions (1–5) show areas of the bulk biofilm matrix from which EDS spectra were derived; green symbols and crosses (i–v) identify type 1 crystal structures from which EDS spectra were derived; orange symbols and crosses (a–e) identify type 2 crystal structures from which EDS spectra were derived. Images were obtained from distinct catheter sections. Cps/Ev: counts per second per electron-volt, keV: kilo-electron-volt. (b) Examples of EDS spectra derived from distinct crystalline structures observed within *Proteus mirabilis* biofilms. Example spectra were derived from regions or structures indicated by associated symbols of corresponding number/letter and on images in part A. (c) Average relative abundance profile of elements detected in EDS spectra derived from the main features of crystalline biofilms observed in ESEM imaging (bulk matrix 1–5, crystal type 1 i–v and crystal type 2 a–e). For each element, data are presented as a proportion of all elements detected in different structures, based on average cps/Ev counts from all spectra analysed.

In order to evaluate the use of additional analytical techniques in combination with ESEM, confirm the visualisation of mineral species known to form within *P. mirabilis* crystalline biofilms (struvite and hydroxyapatite) and gain insight into the composition of the novel type 2 crystals revealed by ESEM, we employed EDS to determine the elemental content of biofilms and specific structures observed (Fig.2). EDS data obtained from scans of the bulk biofilm matrix revealed the presence of elements commonly associated with microbial biomass, expected to be prevalent in the cellular component of biofilms, with increased proportions of C, K and Na compared with both crystal types (Fig.2c). However, elements associated with biofilm mineralisation, in particular hydroxyapatite formation (Ca, P, O), were also well represented in EDS spectra obtained from the bulk matrix.

In contrast, the large electron-dense type 1 crystal structures generated EDS profiles with elevated levels of Mg, N and O compared with other regions of the biofilm analysed, but reduced levels of Ca, confirming these to be struvite crystals (ammonium magnesium phosphates; Fig.2c). Conversely, the novel sheet-like type 2 crystalline forms exhibited EDS profiles more similar to the bulk matrix, but with lower levels of cell-biomass-associated elements (C, K, Na), indicating these to be forms of calcium phosphate (Fig.2). Collectively, these results demonstrate the capacity for ESEM and associated EDS to provide insight into biofilm composition and structure and permit analysis of discrete features in unprocessed biofilms *in situ*.

### ESEM and EDS as a tool for understanding crystalline biofilm development

**Fig 3:**
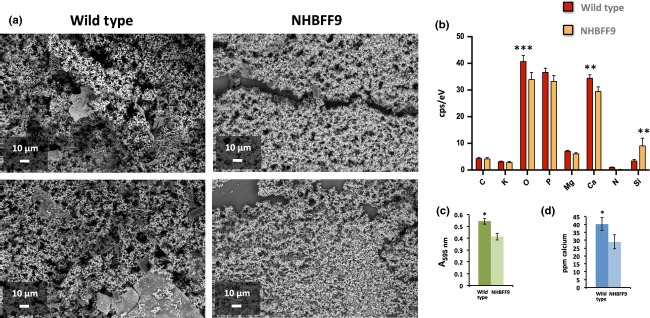
Evaluation of ESEM:ESD for comparative analysis of crystalline biofilm formation by *Proteus mirabilis*. Ten-hour-old biofilms formed on urinary catheters by the wild-type strain B4 and the derivative mini-*Tn*5 mutant NHBFF9 were compared using ESEM and EDS. (a) Example images of wild-type and mutant biofilms. (b) Results of EDS analysis of biofilms showing average cps/Ev counts of elements associated with crystal formation and biofilm mineralisation in wild-type and NHBFF9 mutant biofilms. (c) Comparison of biofilm formation between the wild-type and mutant NHBFF9 on representative sections of urinary catheter, using a modified crystal violet assay. The absorbance of crystal violet solutions obtained after staining of biofilms on catheter sections and elution of retained stain were used as a measure of biofilm formation. (d) Comparison of levels of encrustation, generated by the wild-type and mutant NHBFF9, by flame photometric quantification of calcium. In this case, the same catheter sections analysed using ESEM:EDS were subsequently processed for measurement of calcium by this method. For all charts, error bars show standard error of the mean, and symbols above bars indicate statistically significant differences between the wild type and mutant (*P ≤ 0.05,**P ≤ 0.01, ***P ≤ 0.001). All data were derived from analysis of crystalline biofilms developed in three independent bladder model experiments, with three randomly selected whole fields of view scanned per catheter section for EDS (*n* = 9 fields of view in total).

To evaluate the usefulness of ESEM:EDS as tool for understanding broader aspects of *P. mirabilis* biofilm development, we compared biofilms formed by wild-type *P. mirabilis* and a derivative mini-*Tn*5 mutant designated NHBFF9 (Holling *et al.*, 2014). NHBFF9 is disrupted in a putative efflux system and attenuated in ability to form crystalline biofilms in the bladder model system, but not in ability to elevate urinary pH or persist in bladder models (Holling *et al.*, 2014). Although both wild-type and mutant formed extensive biofilms on catheters, ESEM showed that NHBFF9 biofilms were generally less expansive and of more uniform topology, in keeping with previous characterisation of this mutant (Holling *et al.*, 2014; Fig.3a). Nascent crystal structures commonly observed in wild-type biofilms were also less frequently noted in NHBFF9 biofilms (Fig.3a).

Results of EDS analysis were congruent with these observations and indicated that biofilms formed by the mutant generally contained reduced levels of elements detected in wild-type biofilms (Fig.3b). The exception to this trend was a greater relative abundance of Si in NHBFF9 biofilms. This is in keeping with the visual indication of reduced biofilm formation by this mutant, which results in an increased exposure of the all-silicone catheter surface in many fields of view (Fig.3a and b).

To understand how well these qualitative ESEM:EDS observations represented levels of biofilm formation and encrustation on catheters, we next compared ESEM:EDS results to those obtained from alternative methods for measuring *P. mirabilis* biofilm formation and encrustation (Holling *et al.*, 2014). Encrustation was evaluated using the same catheter sections subject to ESEM:EDS, using flame photometric quantification of calcium, while biomass was quantified on comparable catheter sections using a modified crystal violet assay (Fig.3c and d). Both alternative evaluations of crystalline biofilm formation produced results congruent with the ESEM:EDS analysis, indicating that this can provide useful insights into aspects of crystalline biofilm formation, particularly when utilised in conjunction with representative infection models and molecular genetic approaches (Fig.3b–d).

## Discussion

Here, we show ESEM and associated EDS provide excellent resolution of *P. mirabilis* crystalline biofilms and key crystal structures *in situ* on catheter surfaces. This was in contrast to samples prepared using a common approach for conventional SEM, where the general mineralisation of the biofilm was observed, but larger crystal formations were lost. However, conventional HV-SEM has previously been applied with similar sample processing steps to successfully preserve crystal formations and provide images comparable to those obtained using ESEM in this study (Cox & Hukins, 1989; Winters *et al.*, 1995; Stickler & Morgan, 2008). Differences in strains and species forming the specific biofilms analysed, the conditions under which biofilms were formed, and variations in sample processing are likely to account for the contrast between conventional SEM images generated in this study, and those presented elsewhere. However, in some cases, more specialised techniques for the preservation of *P. mirabilis* crystalline biofilms prior to conventional SEM have been utilised, including the use of the fixatives intended to preserve the extracellular matrix, and critical point drying to minimise introduction of artefacts during dehydration (Stickler, 1996; Stickler *et al.*, 2003).

Nevertheless, even with the application of bespoke methods for sample preparation, the alteration of biofilm features and introduction of artefacts will remain a cause concern. In contrast, ESEM circumvents the requirement for any preprocessing, the specialised equipment and reagents associated with this (particularly where cryo-SEM, freeze-fracturing or critical point drying methods are concerned), and greatly reduces the labour involved in preparation for the samples studied here. This is particularly notable when compared to some of the highly specialised sample preparation approaches developed for the study of *P. mirabilis* crystalline biofilms (Stickler, 1996; Stickler *et al.*, 2003).

The lack of chemical fixation or other processing affords additional advantages to ESEM, in permitting samples to be used with a range of downstream methods directly after viewing, to obtain further data and measurements from the same samples (Bergmans *et al.*, 2005). These include methods for quantification of overall biofilm formation or levels of encrustation, as utilised in the present study (Fig.3), with the recovery and cultivation of cells also possible and demonstrated elsewhere (Bergmans *et al.*, 2005). This maximises the information gained from each sample and permits images providing insight into the overall structure of the biofilm and spatial organisation of key features, to be directly related with other quantitative data or molecular genetic analyses.

ESEM also affords the potential for use of associated analytical techniques during imaging, such as EDS. Although more conventionally applied to smooth surfaces to quantify elemental abundance, EDS has already been used in conjunction with ESEM in a qualitative or semi-quantitative capacity to examine more irregular surfaces, including bacterial biofilms (Spear *et al.*, 2007; MacLean *et al.*, 2008). Of more direct relevance to *P. mirabilis* catheter-associated biofilms is the application of ESEM:EDS to evaluate the composition of initial foundation layers formed on catheter surfaces, after short exposure times to infected urine (Stickler & Morgan, 2008). In contrast, the usefulness of this method in analysing fully formed *P. mirabilis* catheter-associated biofilms *in situ* has not previously been evaluated.

We now confirm EDS to be a viable adjunct to ESEM imaging of these biofilms, providing a useful qualitative analysis of the elemental composition of these unusual microbial communities (Figs2 and 3). EDS spectra derived from various regions of the biofilm are congruent with other studies using conventional SEM and X-ray spectroscopy techniques (Winters *et al.*, 1995; Stickler & Morgan, 2008). In particular, the relatively high proportion of elements associated with calcium hydroxyapatite formation in the bulk biofilm matrix is in keeping with the pervasive mineralisation of mature *P. mirabilis* crystalline biofilms previously reported (Winters *et al.*, 1995; Stickler *et al.*, 2003; Stickler & Morgan, 2008).

In contrast, the sheet-like calcium phosphate structures observed in many ESEM images of the mature biofilms examined in the present study (Figs1 and 2, type 2 crystals), highlight the potential for ESEM to reveal novel features of these biofilms. These sheet-like structures have not previously been described as components of *P. mirabilis* crystalline biofilms, but are reminiscent of crystalline foundation layers, which form on catheters during the very early stages of *P. mirabilis* biofilm formation, and generate similar EDS spectra when analysed during ESEM imaging (Stickler & Morgan, 2008). The similarity of these ‘sheet-like’ crystals to foundation layers raises the possibility that their formation arises simply from damage to the biofilm during catheter sectioning. However, catheter sectioning is also required for visualisation of biofilms by conventional SEM where these sheet-like structures have not been noted. Furthermore, the apparent integration of these structures within the biofilm matrix was also observed in many fields of view, suggesting these features form within developing biofilms.

Alternatively, it is possible that these structures arise as segments of biofilm detach along with underlying foundation layers, during normal development and maturation, followed by incorporation of this material elsewhere in the growing biofilm structure. If so, this would point to a hitherto unappreciated aspect of crystalline biofilm formation in this organism. Regardless of the mechanisms by which these formations arise, it appears the application of ESEM has permitted visualisation of these seemingly delicate structures within mature biofilms, due to the minimal sample handling and processing intrinsic to ESEM, with associated EDS providing information on composition.

The comparison of biofilms formed by the wild-type strain B4, and a derivative mini-*Tn*5 mutant also demonstrated how the ESEM:EDS approach may compliment other methods for evaluating and elucidating the mechanisms underpinning biofilm formation. In this case, ESEM:EDS analysis was able to highlight alterations in biofilm formation, and estimate the relative abundance of elements related to encrustation in the mutant NHBFF9, compared with the wild type. A particularly powerful aspect of this use of ESEM and EDS was the ability to conduct initial visualisation and compositional analysis while the biofilms remained *in situ* on catheter surfaces, but retain the option to subsequently process samples for other measurements.

However, The ESEM and associated EDS methodology is not without limitations, and while this method of imaging and analysis provides an excellent subjective assessment of biofilm formation, results of these endeavours ultimately require validation through quantitative approaches. In addition, our experience of ESEM indicates that in many applications where fully hydrated biofilms are viewed, it may not be possible to achieve levels of magnification comparable to conventional SEM. Nevertheless, overall our results show that ESEM is well suited to analysis of *P. mirabilis* crystalline biofilms and should facilitate a greater understanding of how these structures develop.

## Supporting Information

Fig S1 Illustration of the *in vitro* bladder model system.
