# Supplementary material for: Evaluation of environmental scanning electron microscopy for analysis of Proteus mirabilis crystalline biofilms in situ on urinary catheters
Source: FEMS Microbiol Lett. 2014 May 12;355(1):20–7. doi: 10.1111/1574-6968.12451 (PMC4262002; doi:10.1111/1574-6968.12451)
Supplement: Supplementary file 1 [file fml0355-0020-SD1.pdf]

Supplementary Figure 1

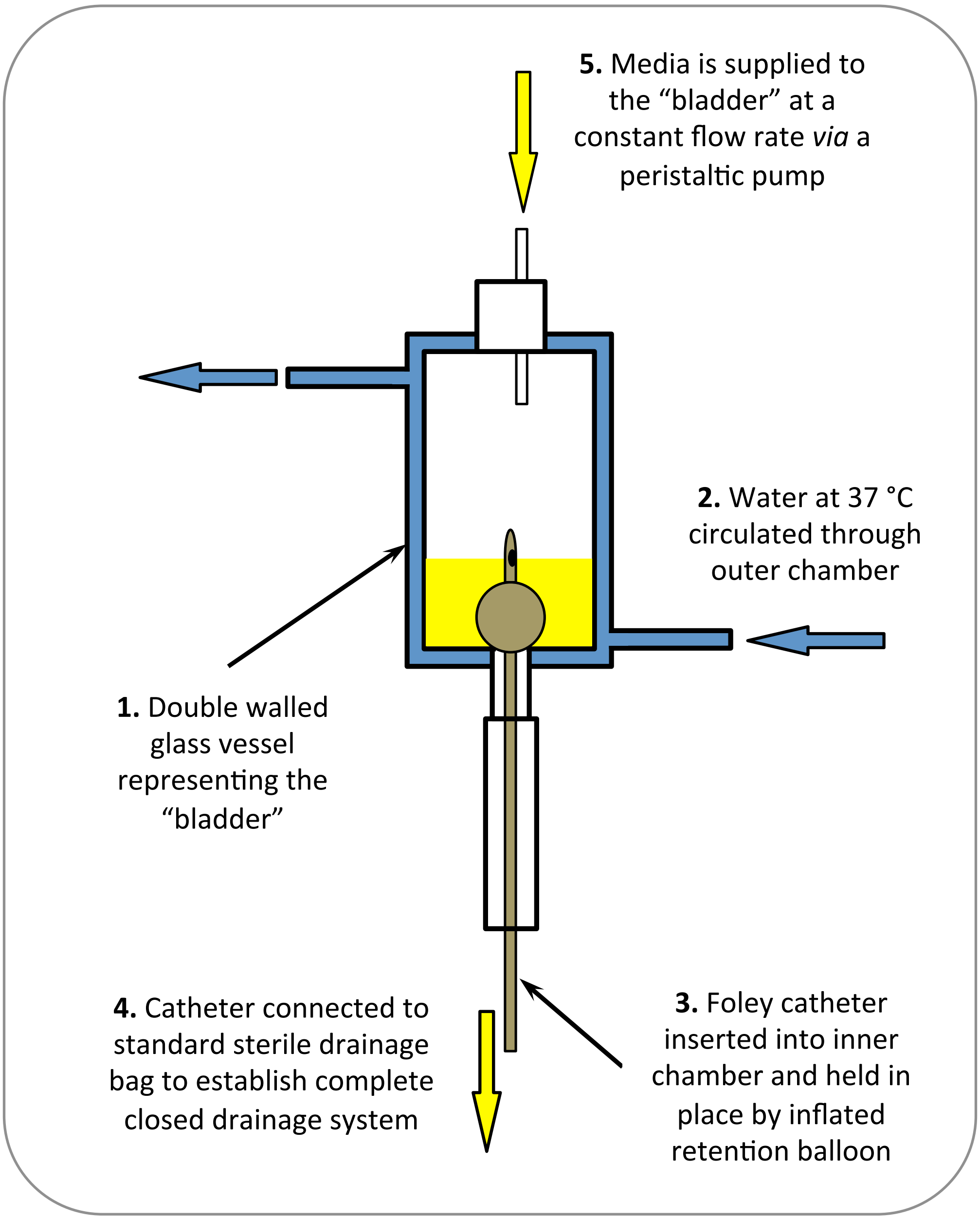

Illustration of the *in vitro* bladder model system (modified from Stickler *et al.* 1999, *Methods Enzymol* 310: 494-501**). 1,2)** Water is circulated through the outer chamber of a double walled vessel to maintain the "bladder" at 37 ºC. **3)** Catheters are inserted into the inner chamber and retention balloons inflated to form a seal and hold the catheter in place. **4)** Catheters are connected to standard drainage bags to form a complete sterile closed drainage system. **5)** Media is supplied to the "bladder" *via* an inlet at the top of the vessel and at a constant flow rate controlled by a peristaltic pump. Test strains are typically inoculated directly into residual media that pools below the catheter eye-hole. Blue arrows indicate water flow though the outer chamber. Yellow arrow indicates urine flow through the closed drainage system.
